# Optimization of quantitative time-resolved 3D (4D) digital subtraction angiography in a porcine liver model

**DOI:** 10.1186/s41747-020-00164-3

**Published:** 2020-07-02

**Authors:** Ece Meram, Gabe Shaughnessy, Colin Longhurst, Carson Hoffman, Martin Wagner, Charles A. Mistretta, Michael A. Speidel, Paul F. Laeseke

**Affiliations:** 1 Department of Radiology, University of Wisconsin-Madison, Madison, WI USA; 2 Department of Medical Physics, University of Wisconsin-Madison, Madison, WI USA; 3 Department of Biostatistics and Medical Informatics, University of Wisconsin-Madison, Madison, WI USA; 4 Section of Interventional Radiology, University of Wisconsin-Madison, Madison, WI USA

**Keywords:** Angiography (digital subtraction), Hepatic artery, Liver, Pulsatile flow, Swine

## Abstract

**Background:**

Time-resolved three-dimensional digital subtraction angiography (4D-DSA) can be used to quantify blood velocity. Contrast pulsatility, a major discriminant on 4D-DSA, is yet to be optimized. We investigated the effects of different imaging and injection parameters on sideband ratio (SBR), a measure of contrast pulsatile strength, within the hepatic vasculature of an *in vivo* porcine model.

**Methods:**

Fifty-nine hepatic 4D-DSA procedures were performed in three female domestic swine (mean weight 54 kg). Contrast injections were performed in the common hepatic artery with different combinations of imaging duration (6 s or 12 s), injection rates (from 1.0 to 2.5 mL/s), contrast concentration (50% or 100%), and catheter size (4 Fr or 5 Fr). Reflux was recorded. SBR and vessel cross-sectional areas were calculated in 289 arterial segments. Multiple linear mixed-effects models were estimated to determine the effects of parameters on SBR and cross-sectional vessel area.

**Results:**

Twelve-second acquisitions yielded a SBR higher than 6 s (*p* < 0.001). No significant differences in SBR were seen between different catheter sizes (*p* = 0.063) or contrast concentration (*p* = 0.907). For higher injection rates (2.5 mL/s), SBR was lower (*p* = 0.007) and cross-sectional area was higher (*p* < 0.001). Reflux of contrast does not significantly affect SBR (*p* = 0.087).

**Conclusions:**

The strength of contrast pulsatility used for flow quantitation with 4D-DSA can be increased by adjusting injection rates and using longer acquisition times. Reduction of contrast concentration to 50% is feasible and reflux of contrast does not significantly hinder contrast pulsatility.

## Key points

- Hepatic arterial blood velocity can be quantified using time-resolved three-dimensional digital subtraction angiography (4D-DSA).
- Imaging and injection parameters significantly affect the quality of 4D-DSA reconstructions.
- Longer data acquisitions lead to the strongest signals for flow quantitation.
- Higher injection rates result in the greatest vascular cross-sectional area.
- Catheter sizes, contrast concentration, or presence of reflux does not hinder signal strength.

## Background

Angiographic techniques currently utilized for treatment planning or endpoint determination rely mainly on anatomic and qualitative hemodynamic information. Time-resolved three-dimensional (3D) digital subtraction angiography (DSA)—*i.e.*, four-dimensional (4D)-DSA—is a recently developed angiographic imaging modality that can provide quantitative information on blood flow within a 3D vascular volume [1]. 4D-DSA has been used for obtaining time-resolved flow information during interventional procedures for cerebrovascular anomalies (*e.g.*, arteriovenous malformations) [2]. Use of 4D-DSA in the thorax and abdomen has been limited primarily by its susceptibility to patient and respiratory motion, a consequence of long data acquisition times and the need for two rotational C-arm acquisitions. Algorithms are being developed to correct for motion-related image degradation during 4D-DSA acquisitions [3]. Improved motion correction will facilitate the use of 4D-DSA during body interventions where characterization of abnormal flow (arteriovenous malformations), flow reduction (transarterial embolizations), or flow restoration (*e.g.*, balloon angioplasty/stent placement for arterial stenosis) is needed.

Angiographic endpoints for liver-directed therapies such as transarterial embolization continue to be somewhat subjective and variable among operators [4]. 4D-DSA can provide *quantitative* information on blood flow and velocity in a vascular volume by leveraging the inherent pulsatility of arterial flow [5]. Quantitative 4D-DSA may provide a better understanding of *in vivo* flow dynamics during hepatic interventional procedures and facilitate the development of rather objective angiographic endpoints. For example, quantitative 4D-DSA was recently shown to be a feasible modality for quantifying hepatic blood flow and characterizing changes in flow during transarterial embolization [6]. However, the quality of the 4D-DSA reconstructions and resultant accuracy of the flow calculations was shown to be dependent on, and positively correlated with, the strength of the contrast pulsatility [7]. Maximizing the strength of pulsatility while maintaining a cross-sectional area with adequate filling would be ideal for 4D-DSA acquisitions. However, data acquisition and contrast injection parameters for 4D-DSA have yet to be optimized to maximize the flow signal. Therefore, this study aimed to investigate the effects of various imaging and injection parameters on quantitative 4D-DSA acquisitions of hepatic vasculature in an *in vivo* porcine model.

## Methods

All procedures were approved by the institutional research animal care and use committee and were compliant with regulatory guidelines (protocol number M005606). Three female Yorkshire cross domestic swine (mean weight, 54 kg) were sedated with an intramuscular administration of 7 mg/kg of tiletamine hydrochloride and zolazepam hydrochloride (Xyla-Ject, Phoenix Pharmaceutical, St. Joseph, Missouri, USA), intubated endotracheally facilitated by 0.05 mg/kg atropine (Phoenix Pharmaceutical, Burlingame, California, USA), and then underwent anesthesia induction and maintenance with 2% inhaled isoflurane (Halocarbon Laboratories, River Edge, New Jersey, USA). After being anesthetized, subjects were placed supine on the bed of a cone-beam computed tomography scanner (Artis Zee, Siemens Healthineers, Erlangen, Germany). Arterial access was obtained via a femoral arterial puncture. A vascular sheath was placed, and a 4 Fr or 5 Fr angled glide catheter was positioned in the common hepatic artery.

### 4D-DSA technique

As previously described [1, 2, 8–11], the 4D-DSA technique consisted of two separate C-arm rotations: the first (mask) rotation without contrast injection and the second (fill) rotation that starts prior to the contrast injection to capture the contrast inflow and time-resolved contrast kinetics. The 4D-DSA images were acquired with 6- or 12-s rotation times over an angular range of 260 degrees. Total data acquisition times (including the mask and fill rotations as well as the mid-acquisition C-arm reset) were 15 and 27 s for the 6- and 12-s rotation times, respectively. Conventional 3D reconstruction was performed to generate a constraint volume after the subtraction of mask and contrast-enhanced fill images. Time-resolved 3D image volumes corresponding to each projection image in the contrast-enhanced rotation were generated by performing constrained back-projection and normalization operations [1].

**Fig. 1:**
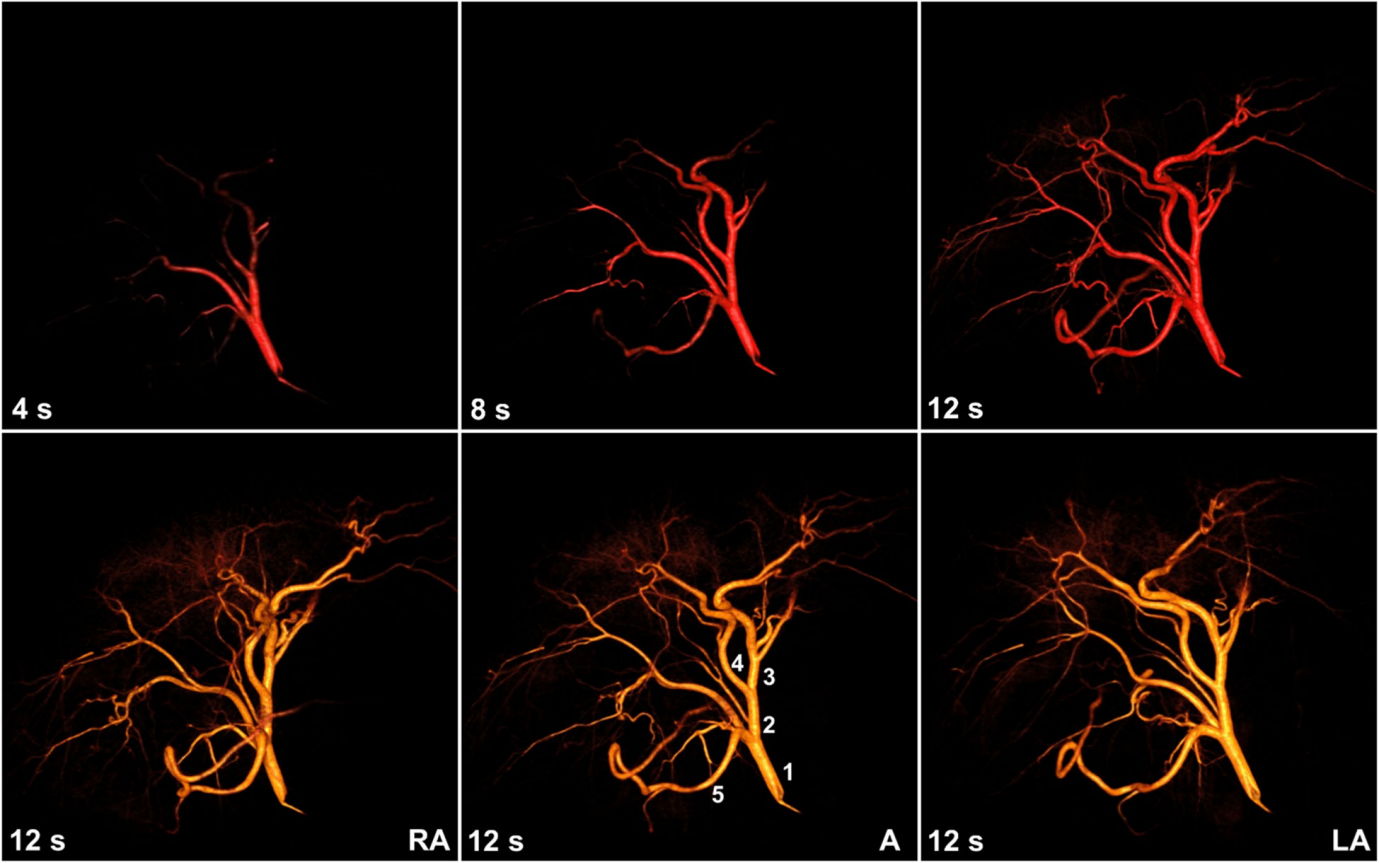
Hepatic 4D-DSA. Upper row shows the hepatic vasculature from an anterior projection with three different time frames at 4, 8, and 12 s; lower row shows the final time frame from three different angles (A, anterior; RA, right anterior; LA, left anterior). Similar images can be obtained for any time frame or angle. The hepatic artery numbering is as follows: 1, common hepatic (1st order); 2, left hepatic (2nd order); 3, left medial (3rd order); 4, left lateral (3rd order); and 5, gastroduodenal (2nd order)

The 4D-DSA acquisitions were performed with the catheter positioned proximally in the common hepatic artery to allow adequate mixing of the contrast and blood as well as to maintain an adequate vessel segment for flow quantitation. Longer acquisition times provide more data with an increased number of cardiac cycles for image reconstruction and flow quantitation; however, they are also more prone to motion and expose the patient to a greater amount of radiation and intravenous contrast. Therefore, two frequently used acquisition times of 6 and 12 s were investigated in this study. The duration of contrast injection was dependent on the duration of the 4D-DSA acquisition. For 6-s and 12-s 4D-DSA acquisitions, 5.5-s and 11-s contrast injections were performed, respectively. The contrast injections were started 0.5-1 s after the initiation of the second gantry rotation in order to capture the inflow of contrast. For all injection protocols, iohexol 300 mgI/mL (Omnipaque 300. General Electric Healthcare, Waukesha, Wisconsin, USA) was used with a max pressure of 800 pound-force per square inch (psi). To minimize the motion-related artifacts in the reconstruction, 4D-DSA images were acquired with respiration suspended. Image reconstruction (Fig. 1) was performed using a 4D-DSA prototype provided by the manufacturer (Siemens Healthineers, Erlangen, Germany).

### Assessment of optimal acquisition parameters

Quantitative flow and velocity measurements can be obtained using a previously validated 4D-DSA based algorithm described by Shaughnessy et al. [7]. This technique relies on a frequency-domain analysis of the pulsatility of the time-attenuation curves as well as 3D geometric information obtained from the iodine signal [7]. Pulsatility refers to the temporal variation in image intensity at each point along a vessel caused by temporal variations in iodine concentration. These concentration variations are a natural result of the mixing of injected contrast agent with time-varying blood flow driven by the cardiac cycle. In this study, 4D-DSA reconstructions were processed using the above algorithm, which also provides a sideband ratio (SBR) metric as a measure of contrast pulsatility in 4D-DSA [7]. The SBR is a proxy for the signal-to-noise ratio of the cardiac pulsatile waveform, and, as such, is a major determinant of flow quantification performance [7]. A higher SBR has been associated with a higher correlation seen between 4D-DSA and intravascular Doppler blood velocities [6]. Given that the objective of the study was to determine factors improving the quality of 4D-DSA reconstructions and flow quantitation, not to actually quantify hepatic arterial flow in a porcine model, SBR, not blood velocity, was the primary endpoint in the analysis.

Total of fifty-nine 4D-DSA examinations were acquired in three subjects with varying injection and acquisition protocols to understand the effect of following parameters on SBR: imaging duration (6 or 12 s), injection rate (1.0–2.5 mL/s), contrast concentration (50% or 100%), and catheter size (4 Fr or 5 Fr). Fifty-nine 4D-DSA examinations corresponded to a total of 299 data points in different arterial segments, which included various combinations of common hepatic (*n* = 59), gastroduodenal (*n* = 68), left hepatic (*n* = 59), left medial (*n* = 56), and left lateral (*n* = 57) arteries depending on the specific anatomy of the individual subjects or limitations with segmentation. Right hepatic arteries were not included as the right-sided porcine hepatic arterial supply is more variable and generally lacks a single dominant right hepatic artery. Given the longer course of the gastroduodenal artery without early bifurcation, for most of the acquisitions (*n* = 30), shorter and longer vessel segmentations were performed for gastroduodenal artery while it could not be segmented in 20 acquisitions.

The primary end point of this experiment was to assess the signal strength of contrast pulsatility (*i.e.*, the SBR metric) with changing injection and acquisition parameters. Data points from different branches of the common hepatic artery were grouped as first-order (common hepatic), second-order (gastroduodenal or left hepatic artery), and third-order (left medial or left lateral artery) branches, and the SBR was evaluated accordingly by taking into account the inherent difference that occurs with branching distally.

Vessel cross-sectional area was selected as a secondary comparison parameter to ensure adequate contrast filling within the vessel with high-quality anatomical detail and accurate flow estimates with 4D-DSA. Therefore, for all vessels in which values for SBR were obtained, the average cross-sectional area was computed with a segmentation algorithm using an automated full width at half maximum approach at local centerline points along the vessel. A segmentation algorithm was chosen to prevent inter-rater differences that could arise with manual segmentation methods. With the remaining parameters being identical, the change in area from 1.0 to 2.5 mL/s injection rate was evaluated to assess the adequacy of filling.

The presence of reflux during injections was also noted for each acquisition to understand the influence of reflux on the SBR.

### Statistical analysis

To estimate the effect of injection rate, scan time, contrast concentration, catheter size, and branch on SBR (on the natural logarithm scale), a linear mixed model was fit to the data using the ‘lme4’ package (V 1.1-1.15) in R (3.4.3) [12]. The model was adjusted for each covariate described above and allowed for interactive effects between scan time and branch number while the individual swine was modeled as a random effect to account for the influence of inter-subject differences on the SBR. Using likelihood ratio tests, it was found that two other candidate models, one which allowed for an interaction effect between injection rate and branch and one which modeled injection rate as categorical factor, were found to not fit the data significantly better than the model described above (*p* = 0.929, *p* = 0.532).

A similar linear mixed model was also fit to analyze the variance of 4D-DSA area. This model adjusted for injection rate, scan time, contrast concentration, catheter size, branch, and included an interaction between injection rate and branch. The individual swine was again modeled as a random effect.

To analyze the effect of assessed reflux on SBR, the linear mixed model described above was refit to the data but with the inclusion of a reflux term. Observations with inadvertent reflux induced via improper catheter positioning or vasospasm around the catheter tip were omitted prior to estimating the model. To assess the role of injection rate, scan time, contrast concentration, catheter size, and branch on reflux, a logistic regression model (allowing for random effects) was estimated.

After estimating each model, 95% semi-parametrically bootstrapped confidence intervals (CIs) were estimated (2000 iterations) and approximate *p* values were calculated using Satterthwaite’s method as implemented in the “lmerTest” package [13].

## Results

Fifty-nine 4D-DSA acquisitions yielded a total of 299 data points. Two 4D-DSA acquisitions (ten data points) were excluded from the analysis due to catheter displacement into the celiac trunk during data acquisition. The analyses were done using the remaining 289 data points. Table 1 shows the types and total number of different parameters that were investigated in this study.

**Table 1:** The acquisition and injection parameters that were changed to investigate their impact on the sideband ratio

Parameters	Investigated values
Acquisition time (s)	6 s (*n* = 71) or 12 s (*n* = 218)
Catheter size (Fr)	4 Fr (*n* = 181) or 5 Fr (*n* = 108)
Injection rate (mL/s)	1.0 (*n* = 63), 1.5 (*n* = 68), 2.0 (*n* = 74), or 2.5 (*n* = 84)
Contrast concentration (%)	50% (*n* = 111) or 100% (*n* = 178)

“*n*” indicates the number of acquisitions under each investigated parameter

**Fig. 2:**
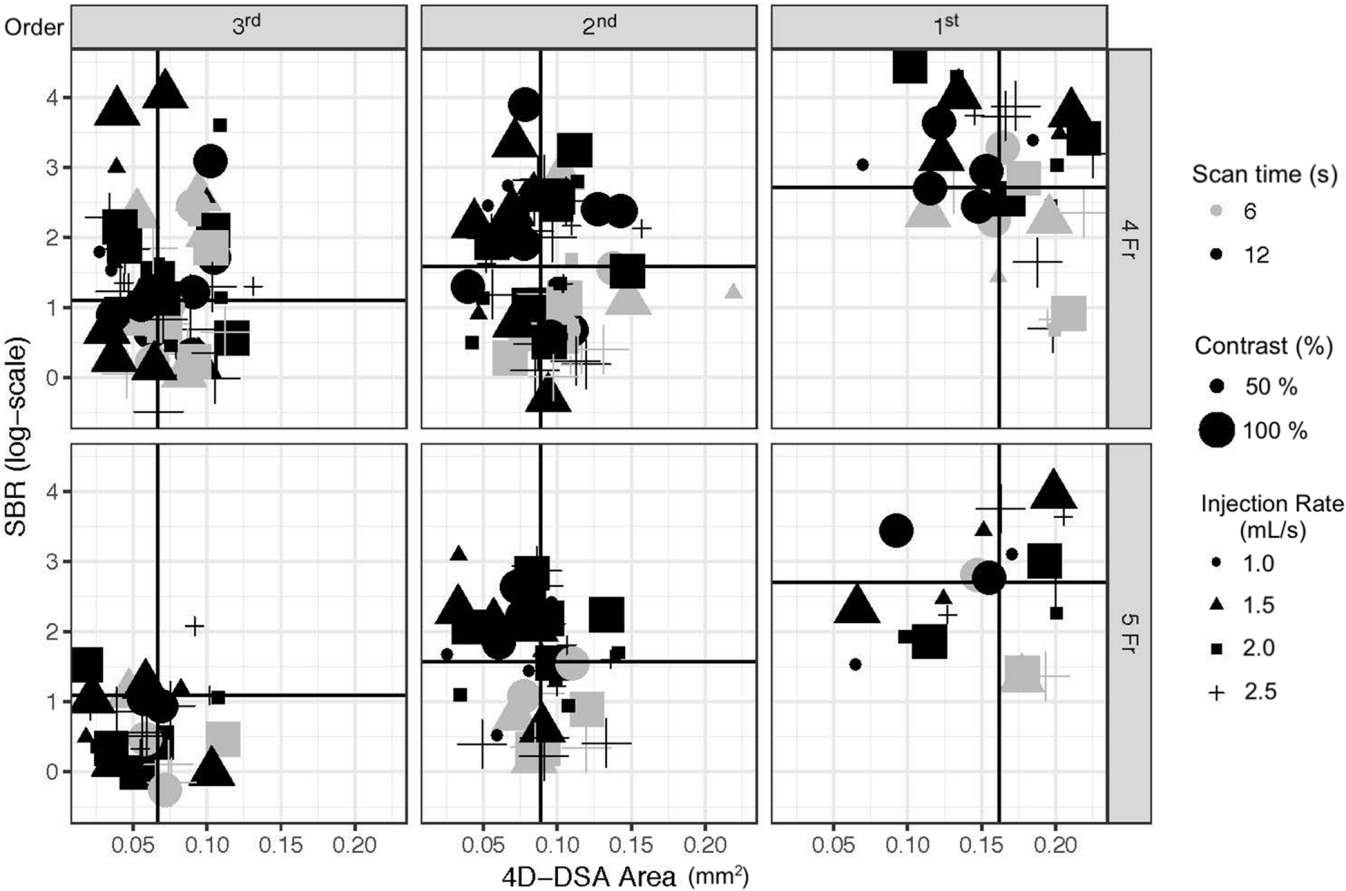
Distribution of the combination of parameters affecting 4D-DSA area and sideband ratio (SBR). The dark x/y intercept lines represent the observed mean SBR and 4D-DSA area values for each branch

The distribution of all 289 data points consisting of different combinations of injection rate, scan time, contrast concentration, and catheter size for the first, second, and third-order branches individually is shown in Fig. 2. A clear trend is not immediately apparent from Fig. 2 with multiple varying parameters. However, linear mixed models that were fit to the data allowed to evaluate the individual effects of investigated parameters. For all linear mixed models, a graphical analysis of the overall model residuals revealed no departure from model assumptions. The overall error, the within-group and the random effects themselves were found to be normally distributed and homoscedastic.

### Assessment of acquisition and injection parameters on sideband ratio

**Fig. 3:**
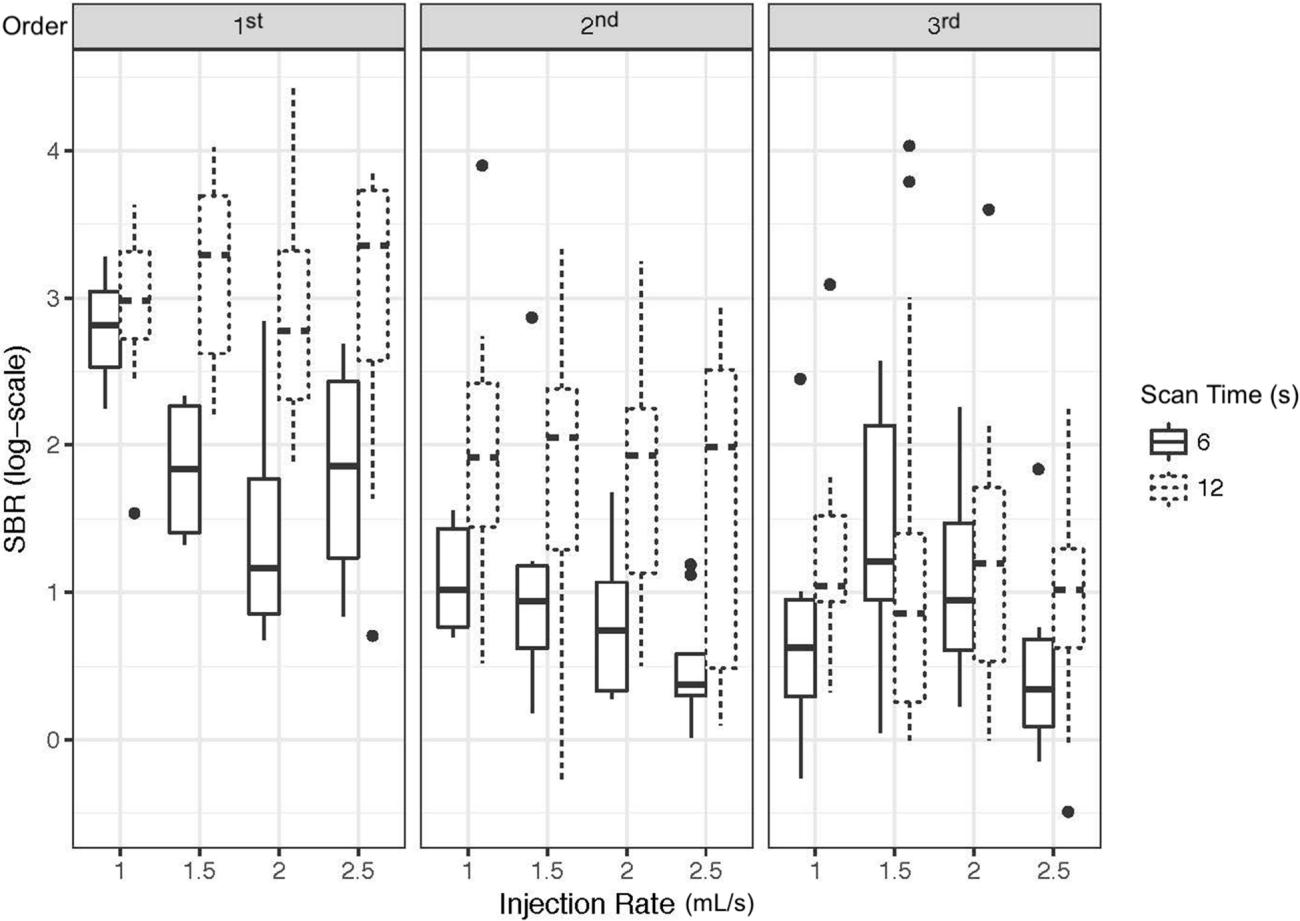
Box-and-whisker plot of observed sideband ratio (SBR) on the natural log scale for 6-s and 12-s scan times with different injection rates in the 1st, 2nd, and 3rd order hepatic arterial branches. Horizontal lines in the box represent median values. Differences in observed SBR between scan times can be seen to decrease in the more distal (3rd order) branches

The mean SBR values and the significance of effect of different acquisition and injection parameters on SBR were summarized in Table 2. As it is shown in Table 3 in further detail, 12-s acquisitions yielded a higher SBR than 6-s acquisitions by a factor of 2.75 (95% CI 1.72–4.35, *p* < 0.001) for the common hepatic artery. While the difference between acquisition times was found to be statistically significant in second-order branches (*p* < 0.0001), a significance was not observed in third-order branches (*p* = 0.297). Compared to the common hepatic artery, SBR in the second-order and third-order branches is estimated to be 61.4% (95% CI 37.8–79.5%, *p* < 0.001) and 63.4% smaller (95% CI 77.2–39.7%, *p* < 0.001), respectively. Overall, a significantly lower SBR was observed in more distal branches. This trend can be seen in Fig. 3. For injection rate (mL/s), when the estimate in log-scale is exponentiated, a one-unit increase was found to be associated with a 19.6% decrease in SBR (95% CI 6.5–31.6%, *p* = 0.007). Neither contrast concentration nor the catheter size were found to be significantly associated with measured SBR (*p* = 0.907 and *p* = 0.063, respectively).

**Table 2:** Mean sideband ratio (SBR) value and standard deviation (SD) for different parameters that were investigated

Parameter	Mean SBR	SD	*p value*
**Scan time (s)**			***< 0.001***
12	10.690	13.148	
6	4.353	4.927	
**Injection rate (mL/s)**			***0.007***
1.0	9.107	9.677	
1.5	10.945	13.527	
2.0	8.816	13.656	
2.5	7.963	10.604	
**Contrast concentration**			*0.907*
50%	9.032	10.602	
100%	9.195	12.799	
**Catheter size (Fr)**			*0.063*
4	10.139	13.381	
5	7.445	8.987	
**Branch order**			***< 0.001***
1st	21.590	17.859	
2nd	7.090	6.722	
3rd	4.913	7.881	

The *p* values were obtained from the linear mixed model for SBR (see Table 3) and reflect the significance of effect for that parameter on SBR

**Table 3:** Linear mixed model summary for sideband ratio (SBR)

Parameter effect on SBR	Estimate	SE	e^estimate^	*p* value
Injection rate	-0.218	0.081	0.804	0.007
Scan time (12 s)	-1.010	0.236	2.746	< 0.001
Branch (2nd order)	-0.952	0.246	0.386	< 0.001
Branch (3rd order)	-1.006	0.245	0.366	< 0.001
Contrast concentration (100%)	-0.011	0.096	0.989	0.907
Catheter (5 Fr)	-0.189	0.101	0.828	0.063
12-s scan: branch (2nd)	-0.211	0.283	0.810	0.457
12-s scan: branch (3rd)	-0.826	0.285	0.438	0.004

Both the regression parameter estimate and the standard error (SE) of that estimate are given on the natural log scale. The exponentiated parameter estimate (e^estimate^) reflects the factor of change in SBR by a unit of change in investigated parameter

### Assessment of 4D-DSA cross-sectional area for vessels

**Fig. 4:**
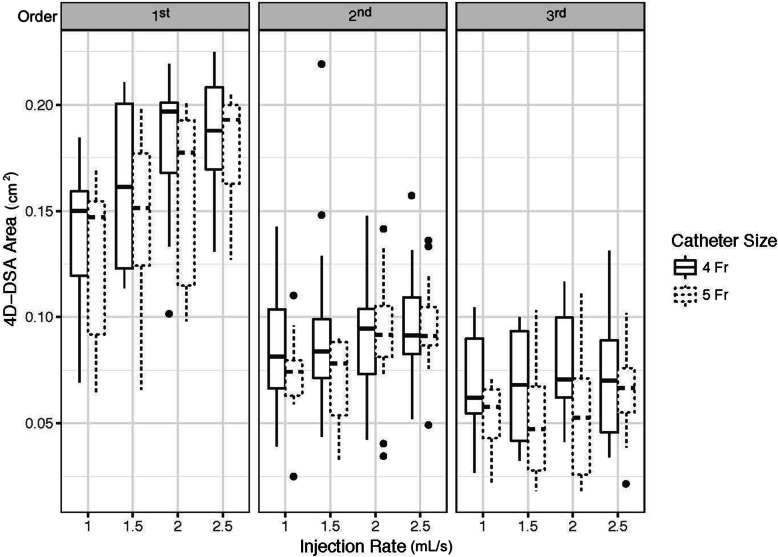
Box-and-whisker plot of observed 4D-DSA area for 4-Fr and 5-Fr catheter sizes with different injection rates in the 1st, 2nd, and 3rd order hepatic arterial branches. Horizontal lines in the box represent median values. Higher injection rates yielded higher 4D-DSA areas and 4-Fr catheters led to a slightly higher 4D-DSA area across all branches

**Fig. 5:**
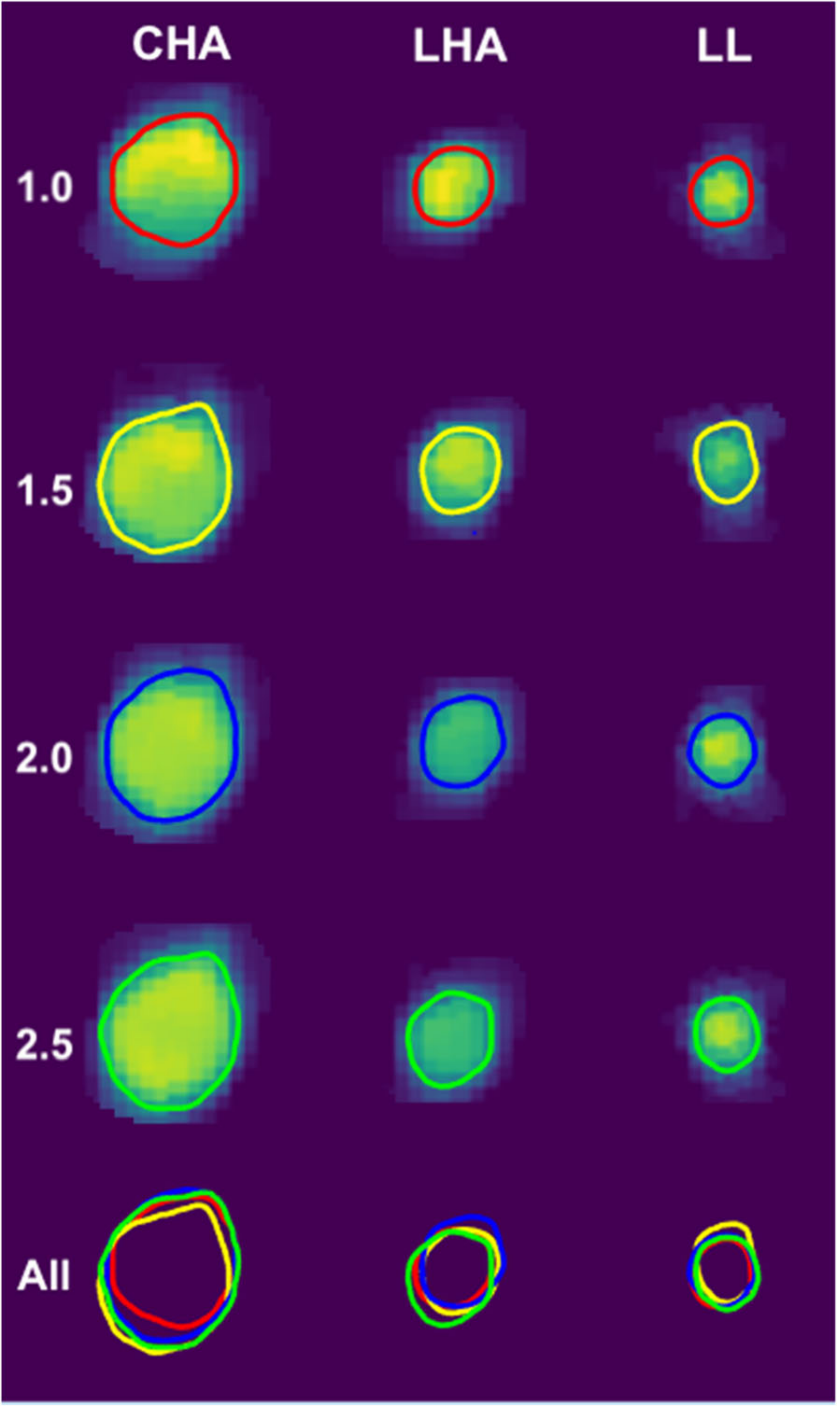
4D-DSA area segmentation of common hepatic artery (CHA, left column), left hepatic artery (LHA, middle column), and left lateral artery (LL, right column) for increasing injection rates from 1.0 to 2.5 mL/s while other parameters were kept identical (12-s scan time, 100% contrast concentration and 4-Fr catheter size). The final row demonstrates the overlapping of all segmentations for different injection rates for the individual vessels

From the model fit to the 4D-DSA area data, it was found that neither contrast concentration nor scan time had a significant effect on estimated area (*p* = 0.633 and *p* = 0.607, respectively). In Table 4, it can also be seen that the rate of injection was positively correlated with 4D-DSA cross-sectional area for the common hepatic artery with an estimated increase in the area by 0.032 cm^2^ (95% CI 0.022–0.042, *p* < 0.001) per unit (1 mL/s) increase in the injection rate. Figure 4 shows the observed 4D-DSA area for 4-Fr and 5-Fr catheter sizes with different injection rates in first-, second-, and third-order hepatic arterial branches. The model also found that both the second- and third-order branches had significantly lower cross-sectional areas compared to the common hepatic artery (*p* = 0.007 and *p* < 0.001, respectively). Finally, injections with a 5 Fr catheter were found to have reduced area by an estimate of 0.008 cm^2^ (95% CI 0.002–0.014) (*p* = 0.009). In Fig. 5, the increase in 4D-DSA area segmentation for increasing injection rates with 12-s scan time, 4-Fr catheter size, and 100% contrast concentration is shown.

**Table 4:** Linear mixed model summary for 4D-DSA arterial cross-sectional area

Parameter effect on area	Estimate	SE	*p* value
Injection rate	-0.032	0.005	< 0.001
Scan time (12 s)	-0.002	0.004	0.608
Branch (2nd order)	-0.033	0.012	0.008
Branch (3rd order)	-0.054	0.012	< 0.001
Contrast concentration (100%)	-0.001	0.003	0.634
Catheter (5 Fr)	-0.008	0.003	0.009
Injection rate: branch (2nd)	-0.022	0.006	< 0.001
Injection rate: branch (3rd)	-0.023	0.007	< 0.001

### Impact of reflux on sideband ratio

**Fig. 6:**
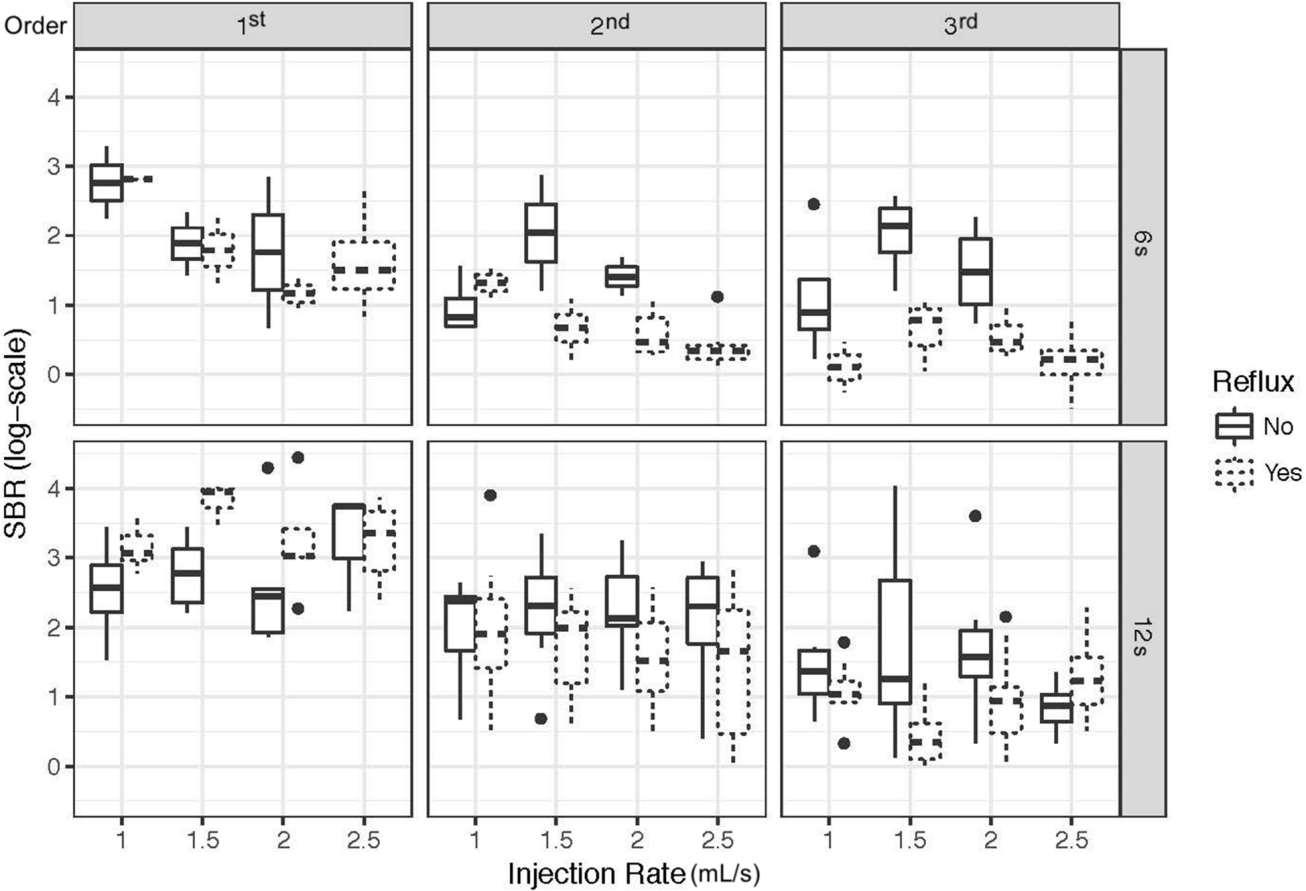
Box-and-whisker plot showing the relationship between reflux, scan time, injection rate, and hepatic arterial branches on observed sideband ratio (SBR). Horizontal lines in the box represent median values. Although reflux was associated with slightly lower SBR values, this effect was not statistically significant

The reflux-adjusted model found no significant effect of the presence of reflux on SBR (*p* = 0.087). Although reflux seems to be more detrimental for the SBR of acquisitions with 6 s scan time than 12 s scan times (as seen in Fig. 6), a model which included an interaction term between reflux and scan time was found not to fit the data significantly better (*p* = 0.106). When the effect of experimental parameters on reflux were investigated using logistic regression, it was found that increasing injection rates (OR 2.59, 95% CI 1.32–5.26, *p* = 0.006) and 100% contrast (OR 4.56, 95% CI 2.14–10.30, *p* < 0.001) resulted in more reflux while 5 Fr catheter (OR 0.09, 95% CI 0.01–0.22, *p* < 0.001) was associated with less reflux. Scan time was found not to be significantly associated with reflux (*p* = 0.677).

## Discussion

Understanding baseline flow states and treatment-related changes in flow is critical when determining if flow-altering catheter-based treatments are indicated and when desired therapeutic endpoints have been reached. Angiographic techniques that are currently used for treatment planning and assessing endpoints provide anatomic and qualitative hemodynamic information. However, given the relative ubiquity of C-arm cone-beam computed tomography in interventional suites, 4D-DSA may allow developing objective *quantitative* physiological endpoints that can be measured and may be easily adopted to interventional practices. This study aimed to investigate the effects of different acquisition and injection parameters that could influence the contrast pulsatility used for quantitation of flow using 4D-DSA images.

The study showed that both injection and data acquisition parameters significantly impact the SBR, which is an estimate of the signal-to-noise and a major determinant in the algorithm that is used for flow quantitation with 4D-DSA. Longer data acquisitions led to higher SBR, possibly due to an increase in the number of cardiac cycles captured and a greater ability to detect the pulsate frequencies captured in the contrast dynamics. Given the significantly higher SBR achieved with longer acquisitions, 12-s acquisitions are suggested for more accurate flow quantitation. However, longer rotational acquisitions are more susceptible to motion artifacts, require larger contrast injections, and results in greater radiation exposure [14]. Therefore, shorter acquisitions may suffice and still be preferable in body parts associated with significant motion or in patients with impaired renal function to minimize the volume of contrast administered.

The study also showed that there were no significant differences between 100% and 50% contrast concentration when all the other parameters were identical. This means that using a contrast concentration of 50% is a feasible option for reducing the amount of contrast used during 4D-DSA acquisitions. An increase in the injection rate was found to negatively impact the SBR, which was observed to be more prominent in the common hepatic artery and for 6-s acquisition time. This could be related to the degree of mixing with higher injection rates, a more turbulent flow near the catheter site, or the overall lower SBR values seen with 6 s acquisition times.

Lastly, a larger cross-sectional area with better filling was observed with higher injection rates. As the area is an important component of flow rate, it is necessary to ensure adequate filling in addition to obtaining a higher SBR (*i.e.*, flow signal) for accurate flow calculations. Given the relatively lower SBRs and larger cross-sectional areas seen with higher injection rates, using an injection rate of 1.5–2.0 mL/s could prevent underfilling while maintaining greater contrast pulsatility.

Reflux of contrast has been previously shown to impact angiographic image quality [15, 16]. As a marker of an excessive amount of contrast being injected, this study also investigated the potential detrimental effects of reflux on 4D-DSA reconstructions. The results demonstrated that reflux occurred more frequently with higher injection rates and 100% contrast concentration. A 5-Fr catheter size was associated with less reflux compared to a 4-Fr catheter, but the 5-Fr catheter also resulted in lower SBR values. This may be due to the smaller size of the porcine hepatic vessels; larger vessels elsewhere in the porcine body or in humans may be able to accommodate a larger catheter size than 4 Fr without losing the contrast pulsatility. Although reflux did yield data with lower SBRs, especially with 6-s acquisition times, there were no statistically significant differences in SBR of the acquisitions with or without reflux. The trend toward a lower flow signal with reflux on shorter acquisitions is likely related to insufficient filling given the overall smaller volume of contrast used during shorter acquisitions. A higher volume of contrast used during longer acquisitions may be able to offset the detrimental effect of reflux on flow signal by ensuring adequate filling of distal branches. It should also be noted that the catheter position might be important in the occurrence of reflux as the more proximal positioning of the catheter tip in the common hepatic artery was observed to reflux more frequently. However, if the flow in the common hepatic artery is of interest, the tip of the catheter should be positioned rather proximally to allow adequate vessel length for contrast mixing and vessel segmentation.

This study had several limitations. It was performed in a porcine model using general anesthesia and breath-holds during data acquisition. A porcine model was selected because it is a commonly used model in interventional research because of the similarity in size and configuration of the hepatic vasculature to humans [17, 18]. Nonetheless, the results of this study need to be validated in clinical patients. The use of general anesthesia was necessary given the animal model, and breath holds were performed because respiratory motion severely degrades 4D-DSA reconstructions. Given many liver-directed therapies in clinical patients are performed under moderate sedation, it can be difficult to achieve adequate breath holds in humans. The long data acquisition during 4D-DSA further compounds this. These limitations are being addressed by motion correction algorithms currently in development [3]. However, neither the impact of respiratory motion on the 4D-DSA reconstructions, nor the ability to correct for it were included in this study. Finally, this study used a pulsatility-based algorithm for flow quantitation. This method may not be as accurate in vessels with lower pulsatility (*e.g.*, distal or post-stenotic arterial branches or veins). However, other techniques not relying on pulsatility (*e.g.*, mean transit time) may be employed in those circumstances.

In conclusion, this study investigated the effect of imaging and contrast injection parameters on the quality of 4D-DSA acquisitions in hepatic arteries using a porcine model. Acquisition parameters significantly impacted SBR, a measure of signal strength required for flow quantitation with 4D-DSA. The strength of contrast pulsatility can be optimized by adjusting the injection rate and using longer acquisition times. Reduction of contrast concentration to 50% is feasible to reduce the amount of contrast administered during 4D-DSA acquisitions. Reflux of contrast does not significantly hinder contrast pulsatility. Further studies are warranted to investigate the applicability of the study results in different organ systems and in clinical patients.

## Data Availability

The datasets used and/or analyzed during the current study are available from the corresponding author on reasonable request.

## Abbreviations

3D: Three-dimensional
4D: Four-dimensional
CI: Confidence interval
DSA: Digital subtraction angiography
OR: Odds ratio
SBR: Sideband ratio
